# Dataset of cheating in childhood: Exploring the link between parental reports of problem behaviors and dishonesty on simulated academic tests

**DOI:** 10.1016/j.dib.2024.111072

**Published:** 2024-10-25

**Authors:** Kaitlyn Wilson, Kanza Batool, Tz-Yu Duan, Catherine Ann Cameron, Kang Lee

**Affiliations:** a Department of Psychology, 38 Dineen Dr, University of New Brunswick, Fredericton, New Brunswick E3B 5A3, Canada; b Ontario Institute for Studies in Education, 252 Bloor Street West, 7th Floor, Toronto, Ontario M5S 1V6, Canada; c Department of Psychology, University of British Columbia, 2136 West Mall, Vancouver, British Columbia V6T 1Z4, Canada

**Keywords:** Dishonesty, Academic integrity, School-aged children, Child Behavior Checklist, Strengths and Difficulties Questionnaire

## Abstract

The present data were reported in the article “Cheating in childhood: Exploring the link between parental reports of problem behaviors and dishonesty on simulated academic tests” (Wilson et al., 2024). It reports the findings from an online study to assess children's cheating behaviors on simulated academic tests. The dataset also contains key demographic information and information from parental reports of their children's behavior on the Child Behavior Checklist and the Strengths and Difficulties Questionnaire. This dataset contains information from 439 North American school-aged children (aged 4-11 years) and their parents. This dataset could be of interest to researchers interested in the consistency of children's cheating behaviors and the associations of cheating to a broader spectrum of childhood behavioral issues.

Specifications Table

**Table:** 

Subject	Psychology
Specific subject area	Developmental and Educational Psychology
Data format	Raw
Type of data	Chart
Data collection	A sample of 439 North American children aged four to 11 years participated in online simulated academic tests of math, knowledge, and motor skills. Cheating on these simulated academic tests was captured using video recordings of the children's behavior while left to complete difficult test questions unmonitored by their parents and the research assistant. Parents of the child participants reported their observations of their child's problem behaviors using the *Child Behavior Checklist* and the *Strengths and Difficulties Questionnaire*. (466 characters)
Data source location	Data collected online with North American participants. Data stored at the University of New Brunswick, the University of Toronto, and the University of British Columbia.
Data accessibility	Repository name: The Open Science Framework (OSF) Data identification number: 10.17605/OSF.IO/NFSMB Direct URL to data: https://osf.io/nfsmb/?view_only=8e67aa254398413daefa9839ea5476f6
Related research article	Wilson K, Batool K, Duan TY, Cameron CA, Lee K. (2024). Cheating in childhood: Exploring the link between parental reports of problem behaviors and dishonesty on simulated academic tests*. Journal of Experimental Child Psychology, 15*(244): 105948. 10.1016/j.jecp.2024.105948

## 1 Value of the Data

• This large dataset provides information about the proportion of North American 4- to 11-year-old children who cheat on simulated academic tests, the consistency of their cheating behavior across different types of tests, and the relationship between parental reports of their children's problem behaviors and their children's cheating behavior on these tests.
• It includes raw data on actually observed cheating behaviors as well as questionnaire responses, allowing researchers to explore cheating tendencies in different types of situations, or to investigate how specific cheating behaviors correlate with specific individual questionnaire responses.
• With its uniquely broad age range, this dataset can be a resource for researchers who are interested in studying the development of cheating behavior during the early school years. It can be used to investigate how cheating inclinations develop with age, helping to identify critical periods for intervention, and potentially to inform strategies for reducing cheating behavior.
• This data set would also be useful to researchers interested in understanding children's cheating behavior in a naturalistic, virtually delivered setting. It could be especially useful for examining how well these findings replicate in traditional in-class academic settings, as well as in virtual learning environments, which have become more prevalent in the post-COVID educational landscape.
• This data set contains large proportions of white (42%) and Asian (42%) participants and may therefore be used as a source for researchers who are interested in cross-cultural differences and similarities in white and Asian children's cheating behaviors.

## 2 Background

Academic cheating, defined as rule-breaking behavior performed to achieve desired outcomes through illegitimate means, is of serious concern to parents and educators given its association with antisocial behaviors and negative long-term outcomes. Despite this concern, limited research examines early childhood academic cheating and its correlates. Specifically, it is unclear whether early childhood academic cheating is an isolated behavior or part of a broader spectrum of childhood behavioral issues. This dataset was collected to test two contradicting theories about childhood cheating [1,2]. The doctrine of specificity theory suggests that cheating is context-specific; thus, children cheat inconsistently across situations. On the other hand, the core character theory posits that (dis)honesty is a stable quality, such that children will cheat consistently across situations. The purpose of this dataset was to inform on this theoretical debate by observing school-aged children's cheating behaviors on simulated academic tasks and examining the relationship between cheating behavior with parental reports of their children's problem behaviors, as measured using the *Child Behavior Checklist* [3] and the *Strengths and Difficulties Questionnaire* [4]. This dataset can inform on whether cheating is situation-specific or a consistent trait by allowing for the analysis of potential relationships between cheating and other behavioral issues*.*

## 3 Data Description

The dataset and codebook are available within the “Data” Excel file through the Open Science Framework (OSF; https://osf.io/nfsmb/?view_only=8e67aa254398413daefa9839ea5476f6). The “Data” file has three sheets. The first, “Codebook”, lists each variable, including those collected from parents using questionnaires, and those observed during the children's cheating tests. Raw data for the individual items are included in addition to the composite variables used in our analyses. Each variable includes a description of how it was collected, as well as response options and response range whenever relevant. Please note that only the full item prompts are available for the *Duke University Religiosity Index*. [5] Item prompts from the *Strengths and Difficulties Questionnaire* [3] and the *Child Behavior Checklist* [4] are not available in our dataset because they are not available in the public domain. However, we do refer to the respective item number from the original scales in the “Variable Label” column in the “Codebook” sheet such that our dataset can be easily reconciled with the original tests. Please contact corresponding author Kaitlyn Wilson (kwilson5@unb.ca) to see the full list of items included.

The “Full Data Set” and “Retained Participants” sheets contain information collected from the parental questionnaires and observations of children's cheating behavior in numerical form. The response label associated with each numerical variable is included in the “Response Options” column in the “Codebook” sheet. The first, “Full Data Set” contains information from the original dataset of 535 participants. Participants missing key variables were excluded (i.e., deleted listwise), such that only those who responded to each of the variables of interest are included in the “Retained Participants” sheet (N=439).

Descriptive analysis of the key findings can be found in the Journal of Experimental Child Studies report [1].

## 4 Experimental Design, Materials and Methods

This data was collected from 439 North American children aged four to 11 years (52% identifying as girls; M_age_ = 7.2, SD_age_ = 1.8) and their parents. Among the children, 42% were identified as Asian, 42% as white, and the rest as Black, Hispanic, Indigenous, or multi-ethnic. Participants were recruited through postings to social media, daycare centers, and word of mouth. We compensated parent participants with $25 Amazon gift cards for their time. Child participants were awarded with completion certificates and gold star stickers, delivered by mail. Parents or legal guardians provided informed written consent for their children's participation in the study and for the audio-visual recording of their children. Children provided verbal assent for their participation.

Children's participation took place across two 1 h Zoom sessions spaced approximately one week apart. While the children were engaged in the research activities, parents completed the scales outlined in Table 1. During the first session, parents completed a demographic questionnaire (i.e., child's gender, age, and ethnicity), the Duke University Religion Index [5], and the Strengths and Difficulties Questionnaire [4]. They completed the Child Behavior Checklist [3] during the second session. These questionnaires were administered virtually using the survey platform Qualtrics and took approximately 15 min each to complete.

**Table 1:** Description of scales completed by parents.

Scale	Number of items	Response options	Range of scores	Cronbach's alpha
*Duke University Religion Index* [5]	5	6-point Likert scale, ranging from ‘rarely or never’ (0) to ‘more than once a day’ (5).	0-25	α = .93
*Child Behavior Checklist* [3]*	33	3-point Likert scale, ranging from ‘not true’ (0) to ‘very true or often true’ (2).	0-66	α = .86
*Strengths and Difficulties Questionnaire* [4]				
Peer Relationship Problems	5	3-point Likert scale, ranging from ‘not true’ (0) to ‘certainly true’ (2)	0-10	α= .50
Conduct Problems	5	3-point Likert scale, ranging from ‘not true’ (0) to ‘certainly true’ (2)	0-10	α= .62
Emotional Symptoms	5	3-point Likert scale, ranging from ‘not true’ (0) to ‘certainly true’ (2)	0-10	α= .69
Hyperactivity-Inattention	5	3-point Likert scale, ranging from ‘not true’ (0) to ‘certainly true’ (2)	0-10	α= .82

⁎ The original *Child Behavior Checklist* [3] consists of 113 items, but for our research, parents only completed a selection of 33 items to reduce redundancy, exclude age-inappropriate or potentially triggering items, and minimize the time commitment required of parents. The items selected for inclusion were numbers 1, 4, 8, 10, 11, 12, 13, 17, 25, 26, 27, 28, 34, 36, 38, 39, 41, 43, 48, 61, 62, 63, 64, 67, 72, 78, 79, 80, 81, 82, 90, 101, 106.

Meanwhile, we conducted three sets of tests that simulate academic testing scenarios to measure cheating in children. These tests were adapted from existing paradigms based on the work of Hartshorne and May (1928) [6] which have been successful at eliciting cheating behavior in school-aged children. Each of the two online sessions contained one math test, one knowledge test, and one motor-skill test, as described in Table 2. Thus, in total, children had six opportunities to cheat across the two sessions. We rewarded children with gold stars and an A grade for their successful completion of each of the tests. Both gold stars and a letter grade were awarded as we expected the gold stars to be more motivating for the younger children and the academic grade to be more motivating for the older children but both potentially simulating academic-typed incentives. These tests were standardized to approximate academic testing as closely as ethically possible to elicit similar decision-making processes to those involved in academic cheating.

**Table 2:** Tests used to measure children's cheating behavior.

Test (Session)	Description of Test	Coding of Cheating
Math Test: Counting (1)	Each question involved four boxes holding varying numbers of fruit (e.g., four bananas, three pears, five strawberries, and three oranges). The children were asked to name the fruit of the greatest number in four simple practice rounds. The final round contained too great a number of fruit icons for any child to reasonably count.	Coded as intentionally moving the object or peeking over it to see the correct answer in the research assistant's absence.
Knowledge Test: Sounds (1)	Each question played the sound for the child to name. The first four practice rounds involved easily identifiable sounds (e.g., a car horn, a dog bark). The final round involved the sound of a rarely heard animal (e.g., fox)	Coded as intentionally moving the object or peeking over it to see the correct answer in the research assistant's absence.
Motor Skills Test: Balancing (1)	Conducted in a large, safe space with the camera capturing their child's entire body. Children were instructed to follow three rules: 1) “Stand only on one leg,” 2) “keep your eyes closed while you balance,” and 3) “if you put your foot down before the timer is complete, do not try to balance again.” Children practiced balancing on one foot and completed two simple practice rounds. The final round involved balancing for one minute with eyes closed.	Coded as putting a foot down, opening of the eyes, or trying to balance again after an unsuccessful trial.
Math Test: Pattern (2)	Each question displayed an arithmetic pattern of images and the child was asked to identify which icon came next in the pattern. The four practice rounds contained simple patterns for the children to complete. The final round contained a non-sensical pattern that would be impossible to complete.	Coded as intentionally moving the object or peeking over it to see the correct answer in the research assistant's absence.
Knowledge Test: Images (2)	Each question displayed a zoomed in image for the child to identify (e.g., zebra stripes). The final question contained an image that would be very difficult to identify (e.g., the gills of a mushroom).	Coded as intentionally moving the object or peeking over it to see the correct answer in the research assistant's absence.
Motor Skills Test: Ball Throw (2)	Conducted in a large area with five crumpled-up paper balls, a large bowl, and a long, thin object to form a line. Children were informed of the rules: 1) “Do not to step beyond the line”, 2) “Do not rethrow an unsuccessful ball”, and 3) “Do not to move the line.” Participants practiced throwing balls over three practice rounds. Each round was incrementally more difficult, beginning with a distance of one foot, then moving the bowl incrementally after each successful practice round. During the final round, participants were asked to throw two paper balls in the bowl from approximately four feet.	Coded as moving over the established line, trying to rethrow an unsuccessful ball, or moving the line.

**Fig. 1:**
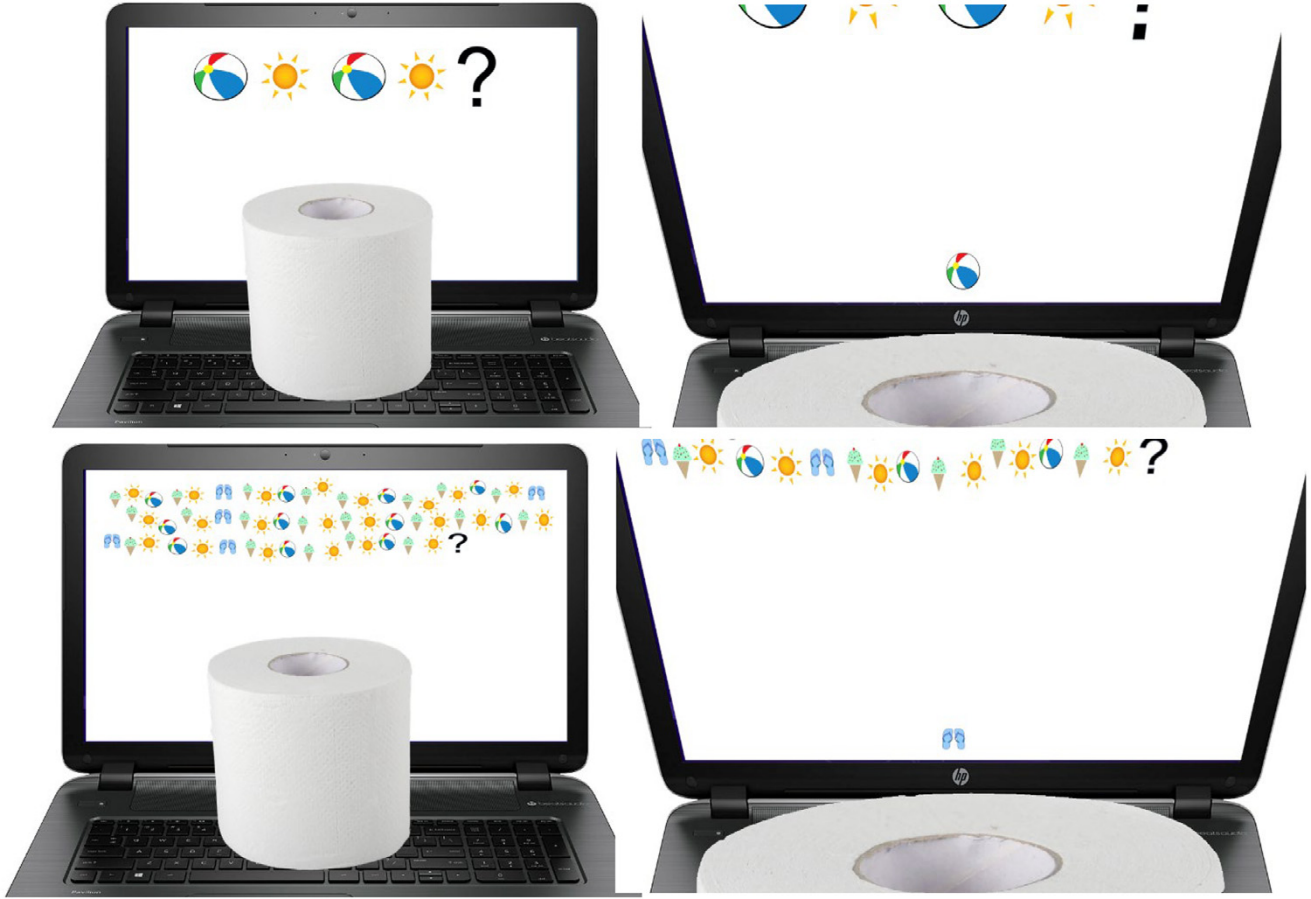
Examples of a first (top row) and fifth (bottom row) round of a test from a seated (left column) and standing (right column) position. The obstructing object was set up by the parent with the assistance of the research assistant to ensure that the children could not see the correct answer at the bottom of the screen without cheating. This figure was retrieved from Wilson et al. (2024) [1] and is licensed under a CC BY 4.0 license.

For each test, we interacted individually with children over Zoom while screen capture software video-recorded children's behaviors throughout the two sessions. Parents received instructions prior to the session for setting up for the math and knowledge tests by positioning a light object (e.g., a tissue box or toilet paper roll) to cover the bottom portion of the screen containing the correct answer. Parents made themselves available to set up the obstructing object during the sessions and aided the research assistant to trial the setup such that the children would not be able to see the correct answer while sitting down and would only be able to see if the child were to move or peek over the object (see Fig. 1). Piloting the set up with the assistance of parents also allowed the research assistant to confirm that cheating behavior would be readily detectable through the audio-visual recording. Parents then left the room to avoid influencing their child's behavior.

For each math and knowledge test, the correct answer for each trial was presented at the bottom of the computer screen and obstructed from view by the object until the researcher permitted the children to check the correct response. In cases where children had siblings taking part, they were given different final questions to prevent them from sharing answers.

For each of the six tests, the researcher engaged children in simple practice rounds to familiarize them with the rules of the activity. For each successful round, they were awarded a gold star. The fifth and final round was intentionally made extremely difficult, so it would be unlikely for children to complete the task successfully. Children were awarded a fifth gold star and an "A" letter grade for completing this final round successfully; otherwise, they were reminded that there would be more opportunities to win gold stars and were left with a "B" letter grade. The tests had varying levels of difficulty based on the child's age: a simple version was given to children aged six and below, a slightly more challenging version was given to children aged 7-8, and the most difficult version was given to those aged nine and above. Specifically, the practice rounds of each test were developmentally appropriate to maintain interest from the participants, while still being simple enough for the children to complete. For example, the math game in the first session (described in Table 2) required participants to identify which of four boxes held the greatest number of fruits. The youngest children received a version with fewer fruit in each box (i.e., no more than five), while the oldest children received a version that required them to count a greater number of fruit (i.e., up to 10). Regardless of the version, this final round was always very difficult for children such that they would not be able to successfully complete the test without cheating. Before leaving the children's view from the computer, the research assistant reminded children of the rules and of the opportunity to be awarded an A grade and gold stars. The children's video-recorded behavior in the research assistant's absence was used to code for the presence of cheating, as defined in the third column of Table 2. For example, cheating on the math and knowledge tests was coded as the child intentionally moving or peeking over the obstructing object. The placement of the obstructing objects was standardized with the help of the parents to ensure that the children could not see with correct answer without cheating, and that intentionally moving or peaking over the object would be easily identifiable as cheating in the video recording.

Parents and their children were debriefed by the research assistant following the protocols approved by our Research Ethics Committees.

## Limitations

This dataset comes with certain limitations, particularly regarding the parental report measures. While our internal consistency measures approximate those established for the scale (α = .59, .71, and .74 for Peer Relationship Problems, Conduct Problems, and Emotional Symptoms, respectively [7]), several of the subscales of the *Strengths and Difficulties Questionnai*re [4] fell below adequate reliability (α≥ .7). Specifically, we obtained internal reliability values of *α* = .50, .62, and .69 for Peer Relationship Problems, Conduct Problems, and Emotional Symptoms, respectively.

Additionally, the administration of the *Child Behavior Checklist* [3] to a non-clinical sample produced relatively low variability, as most parents reported few to no behavioral problems on this scale.

Finally, while the simulated academic tests were standardized to approximate school settings as closely as possible, virtual administration of these tests from home may not have elicited cheating behaviors in the same way as they might occur in a classroom environment, thus potentially limiting the external validity of this dataset. (158 words)

## Ethics Statement

The authors received informed, written consent to participate in the study from children's parents or guardians and received verbal assent from the child participants. Parents and their children participated voluntarily and could withdraw themselves and their data from the study at any time. This study was approved by the research ethics boards at the University of New Brunswick (REB #2020-170), the University of Toronto (REB #25664), and the University of British Columbia (REB #H20-03126).

## CRediT authorship contribution statement

**Kaitlyn Wilson:** Data curation, Formal analysis, Methodology, Project administration, Visualization, Writing – original draft, Writing – review & editing. **Kanza Batool:** Data curation, Formal analysis, Project administration, Supervision, Writing – original draft. **Tz-Yu Duan:** Conceptualization, Formal analysis, Methodology, Project administration, Supervision, Visualization, Writing – original draft. **Catherine Ann Cameron:** Conceptualization, Data curation, Formal analysis, Funding acquisition, Investigation, Methodology, Project administration, Supervision, Writing – original draft, Writing – review & editing. **Kang Lee:** Conceptualization, Data curation, Formal analysis, Funding acquisition, Investigation, Methodology, Project administration, Supervision, Visualization, Writing – original draft, Writing – review & editing.

## Data Availability

OSFCheating in Childhood: Data Exploring the Link between Parental Reports of Problem Behaviors and Dishonesty on Simulated Academic Tests (Original data).
